# Comparison of Correlation between 3D Surface Roughness and Laser Speckle Pattern for Experimental Setup Using He-Ne as Laser Source and Laser Pointer as Laser Source

**DOI:** 10.3390/s22166003

**Published:** 2022-08-11

**Authors:** Suganandha Bharathi Jayabarathi, Mani Maran Ratnam

**Affiliations:** 1 Faculty of Engineering and Computer Technology, AIMST University, Semeling, Bedong 08100, Kedah, Malaysia; 2 School of Mechanical Engineering, Engineering Campus, Universiti Sains Malaysia, Nibong Tebal 14300, Penang, Malaysia

**Keywords:** surface roughness, speckle pattern, milled surface, non-contact, optical technique

## Abstract

Correlation between 3D surface roughness and characteristic features extracted from laser speckle pattern was done using an inexpensive laser pointer and a digital single lens reflex (DSLR) camera in previous research work. There had been no comparison work done between the experimental setup which uses a laser pointer, which has a diode laser as the laser source, and the experimental setup, which uses a He-Ne laser as the laser source. As such, in the current work, a comparison study between two experimental setups was carried out. One experimental setup was using a He-Ne laser, spatial filter, and charged coupled device (CCD) camera, while another experimental setup was using a laser pointer and DSLR camera. The laser beam was illuminated at angles of 30°, 45°, and 60° from the horizontal. When a laser beam falls on the surface, the beam gets scattered, and the scattered beam undergoes interference and produces speckle patterns which are captured using a camera. Using a Matlab program, the gray level co-occurrence matrix (GLCM) characteristic features, such as contrast (GLCM), correlation (GLCM), energy (GLCM), entropy (GLCM), homogeneity (GLCM), and maximum probability, and non-GLCM characteristic features, such as mean, standard deviation (STD), uniformity, entropy, normalized R, and white-to-black ratio (W/B), were extracted and correlated with 3D surface roughness parameters. The coefficient of determination (R^2^) was determined for each case. Compared to the setup using a laser pointer, the setup using a He-Ne laser gave better results. In the setup using the He-Ne laser, there were correlations with a coefficient of determination R^2^ ≥ 0.7 at illumination angles of 30°, 45°, and 60°, whereas in the setup using a laser pointer, there were correlations with R^2^ ≥ 0.7 at illumination angles of 30° and 45°. Mean characteristic features had more correlations with R^2^ ≥ 0.7 in the case of the angle of illumination of 45° (7 out of 36 correlations) and 60° (11 out of 82 correlations), while R-normalized characteristic features had more correlations with R^2^ ≥ 0.7 in the case of the angle of illumination of 30° (9 out of 38 correlations) for the setup using the He-Ne laser. Correlation (GLCM) had more correlations with R^2^ ≥ 0.7 in the case of the setup using a laser pointer (2 out of 2 correlations for illumination angle of 30°, and 4 out of 19 correlations for an illumination angle of 45°). Roughness parameters *S_a_* and *S_q_* had more correlations with R^2^ ≥ 0.7 for an illumination angle of 30° (1 out of 2 correlations each), and *S_p_* and *S_z_* had more correlations with R^2^ ≥ 0.7 for an illumination angle of 45° (4 out of 19 correlations each) in the case of the setup using a laser pointer. The novelty of this work is (1) being a correlation study between 3D surface roughness and speckle pattern using a He-Ne laser and spatial filter, and (2) being a comparison study between two experimental setups on the correlation between 3D surface roughness and speckle pattern.

## 1. Introduction

Surface roughness refers to finely spaced irregularities formed during the machining process [1]. Surface roughness influences mechanical part parameters such as fit, wear resistance, fatigue strength, contact stiffness, vibration, and noise. These variables have an impact on a product’s service life and dependability [2]. As a result, surface roughness measurement is critical in the production process. Surface roughness can be measured with either a contact or non-contact method. The stylus probe is a widely used contact method in the industry. This technology, however, has some drawbacks, including a long measuring time and the stylus accuracy being dependent on its tip radius, which means it may not be able to reliably detect surfaces with crevices smaller than the stylus tip [3].

On the surface of soft materials, the stylus tip could cause a scratch. White light interferometers [4,5], the focus variation method [6], and confocal microscopy [7] are the most common non-contact methods for assessing 3D roughness parameters currently accessible. Machine vision has a high measurement efficiency, a big data acquisition capacity, high measurement accuracy, and flexibility [2].

Statistical properties [8,9,10], wavelet transform [11], Tsallis threshold [12], neural network [3], gray level co-occurrence matrix (GLCM) [10,13], lacunarity [14], spectral speckle correlation [15,16,17,18], and contrast [19,20] are some of the techniques used in vision methods for correlating characteristics features with surface roughness. Profile roughness parameters and areal roughness parameters are two types of surface roughness parameters. The profile roughness parameters are also referred to as two-dimensional or 2D roughness parameters, whereas the areal roughness parameters are referred to as three-dimensional or 3D roughness parameters [8,21,22,23].

Current vision approaches extract characteristics to correlate with 2D roughness parameters [9,13,20,24,25]. However, machined surfaces are 3D in nature and, hence, 3D surface roughness parameters should be measured [8]. Jayabarathi and Ratnam [10] used characteristic features extracted from laser speckle patterns for correlation with 3D surface roughness. The researchers used a laser pointer instead of a He-Ne or diode laser which are widely used in the research works involving speckle patterns. However, no literature is available on how the results obtained with the setup used by Jayabarathi and Ratnam [10] compared to the experimental setup used by other researchers. It is essential to compare the two different setups, as replacing the He-Ne laser and spatial filter setup with a laser pointer simplifies the experimental setup, and is also inexpensive compared to the commercial He-Ne laser. This is a continuation of the work of Jayabarathi and Ratnam [10], where a comparison is carried out between an experimental setup using a laser pointer and digital single lens reflex (DSLR) camera and an experimental setup using a He-Ne laser, spatial filter, and charged coupled device (CCD) camera. In addition to that, there is no publication, to the authors’ best knowledge, on any research work involving a correlation study between 3D surface roughness and characteristic features extracted from laser speckle pattern where the experimental setup consists of a He-Ne laser, spatial filter, and CCD camera. Moreover, no comparison studies were done between the experimental setup using the He-Ne laser and the experimental setup used by Jayabarathi and Ratnam [10].

## 2. Materials and Methods

### 2.1. Sample Preparation

**Figure 1:**
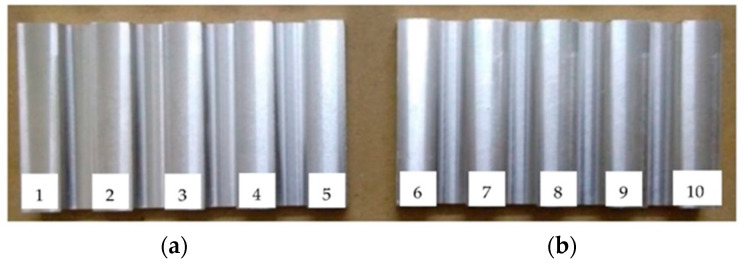
(**a**) Sample 1 and (**b**) Sample 2 with numbering [10].

Two samples with five surfaces each were machined on a CNC 5-axis milling machine (DMU 40 monoBLOCK by Deckel Maho, Bielefeld, Germany) with a four flute high-speed steel (HSS) end mills cutter with a diameter of 12 mm. One of the most essential advantages of high-speed steel is its ability to cut through materials at high speeds. Because of the alloy’s unique combination of hardness, wear resistance, and high-temperature characteristics, one may make use of this benefit. HSS tools are also less expensive than carbide tools, making them an excellent choice for high-mix, low-volume applications. Sample 1 and sample 2 are the names of the two reference samples. Figure 1 shows the two machined samples. Each sample has 5 surfaces, and the surfaces are labeled 1 to 10.

Each surface was machined at different machining parameters, and using the Alicona Infinite Focus Microscope by Bruker Alicona, Austria, the following 3D surface roughness parameters [26] of each surface were measured. The roughness values are tabulated in Table 1 [10].

- Arithmetic mean height (*S_a_*);
- Root-mean-square height (*S_q_*);
- Maximum peak height (*S_p_*);
- Maximum valley depth (*S_v_*);
- Maximum height (*S_z_*);
- Ten point height (*S_10z_*);
- Skewness (*S_sk_*);
- Kurtosis (*S_ku_*);
- Root-mean-square gradient (*S_dq_*);
- Developed interfacial area ratio (*S_dr_*).

### 2.2. Experimental Setup 1

**Figure 2:**
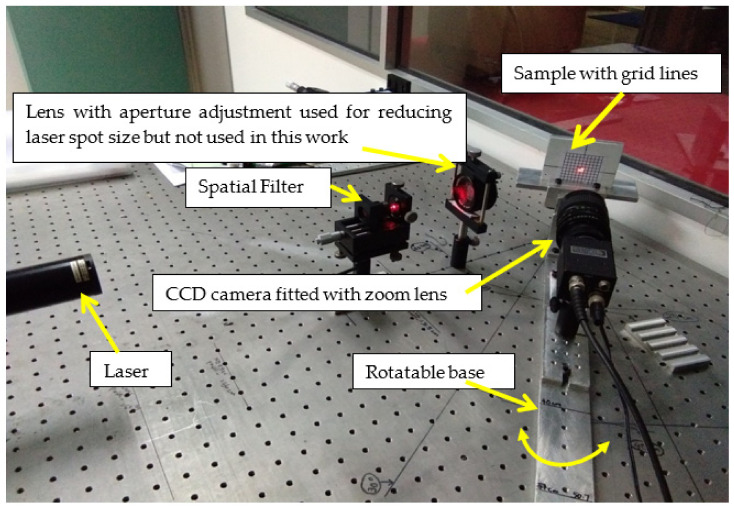
Experimental setup 1.

Figure 2 shows experimental setup 1. Experimental setup 1 consisted of a He-Ne laser used as the laser source; the laser beam was cleaned and expanded using a spatial filter setup. The laser beam then fell onto the milled surface and was scattered, and this scattered beam underwent interference which resulted in a laser speckle pattern.

**Figure 3:**
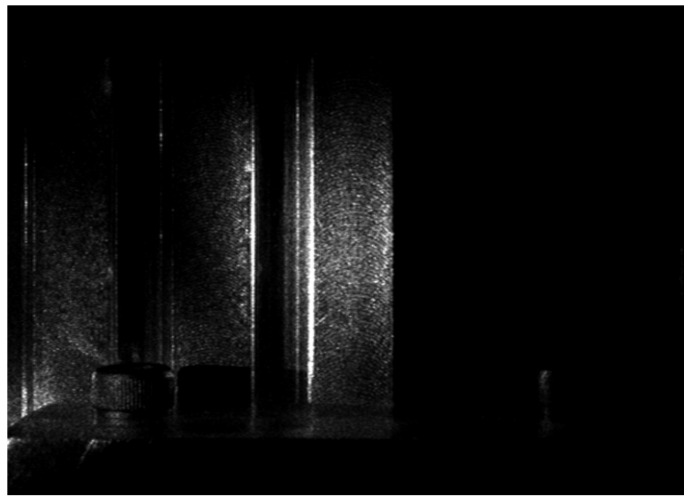
Laser speckle pattern from experimental setup 1.

This laser speckle pattern was captured using a CCD camera (JAI CV-M50, JAI, Japan) fitted with a TV zoom lens (1:1.2/12.5–75) and an extension tube of size 20 mm. The viewing direction of the CCD camera was placed normal to the machined surfaces. The images were stored in Tag Image File Format (TIFF). The CCD camera and the machined surface together could be rotated. Experiments were conducted for the combination of illumination angle of the laser beam of 30°, 45°, and 60°, and camera aperture sizes (*f*-number) of 4, 5.6, 8, 11, and 16. The CCD camera was fixed relative to the machined surface. Figure 3 shows the laser speckle pattern from experimental setup 1 for one of the machined surfaces. Laser speckle images for each of the 10 machined surfaces were captured. The laser speckle pattern image was 768 × 576 pixels and saved in TIFF format. 

### 2.3. Experimental Setup 2

**Figure 4:**
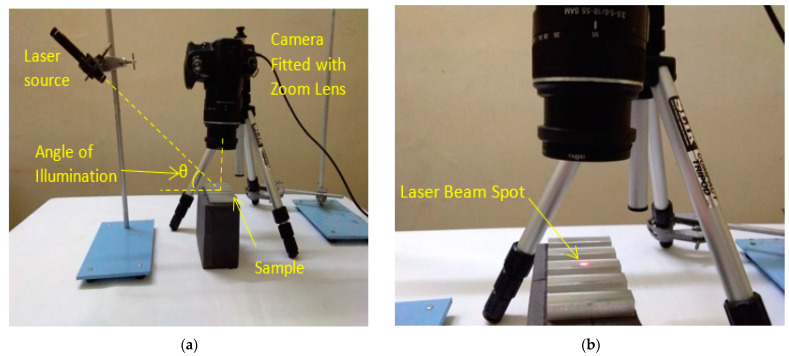
(**a**) Experimental setup 2 and (**b**) close-up view of the sample illuminated by laser beam [10].

**Figure 5:**
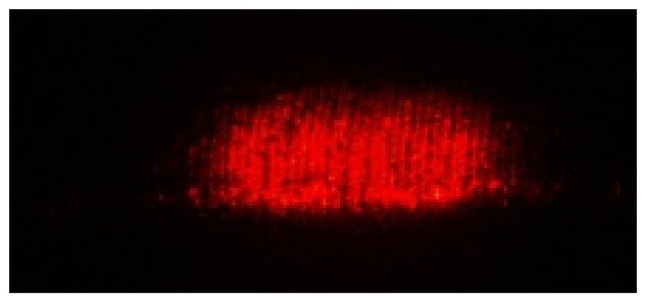
Laser speckle pattern [10].

Because this is a continuation of the work reported by Jayabarathi and Ratnam [10], experimental setup 2 was the same as that previously published. Experimental setup 2 is shown in Figure 4. As illustrated in Figure 4a,b, a laser beam from a commercial laser pointer (LX1 by Legamaster, Ahrensburg, The Netherlands) with a 5 mm diameter red laser dot, wavelength between 630 and 680 nm, and maximum output less than 1 mW, was focused onto the sample at the necessary angle. The scattered beam underwent interference, resulting in a laser speckle pattern. The laser speckle pattern image of size 3872 × 2592 pixels was acquired using a Sony Camera DSLR-A230, Japan (image resolution of 3872 × 2592 pixels) paired with an 18-55 mm smooth autofocus motor (SAM) Sony lens and close up +8 lens, as illustrated in Figure 5. The lens was set to manual focus with a focal length of 55 mm. The experiment apparatus was covered with black fabric to ensure that no external lighting was present.

The laser speckle patterns obtained from all the ten milled surfaces were captured at various combinations of the *f*-number and shutter speed setting of the camera. The illumination angle of the laser pointer used was 30°, 45°, and 60°, the *f*-number used were 8, 16, 22, and 32, while the shutter speeds used were 1/50, 1/100, 1/200, and 1/400 s, resulting in 48 speckle pattern images. All experimental work and analysis were carried out one time only.

### 2.4. Characteristic Features Extraction

**Figure 6:**
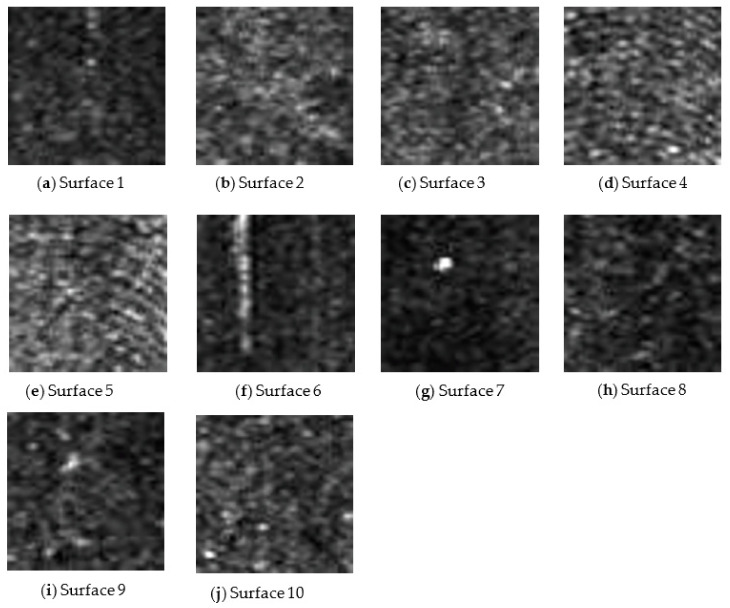
(**a**–**j**) The grayscale images of the laser speckle pattern image for each surface at an illumination angle of 45°, and *f*-number of 8.

In the case of experimental setup 1, using MATLAB 2021a software, the speckle pattern image was cropped to a size of 51 × 51 pixels (maximum possible size) and converted from an RGB image to a grayscale image. Figure 6 shows the cropped grayscale image for the speckle pattern obtained for surfaces 1 to 10 at an illumination angle of 45° and *f*-number of 8 for experimental setup 1.

**Figure 7:**
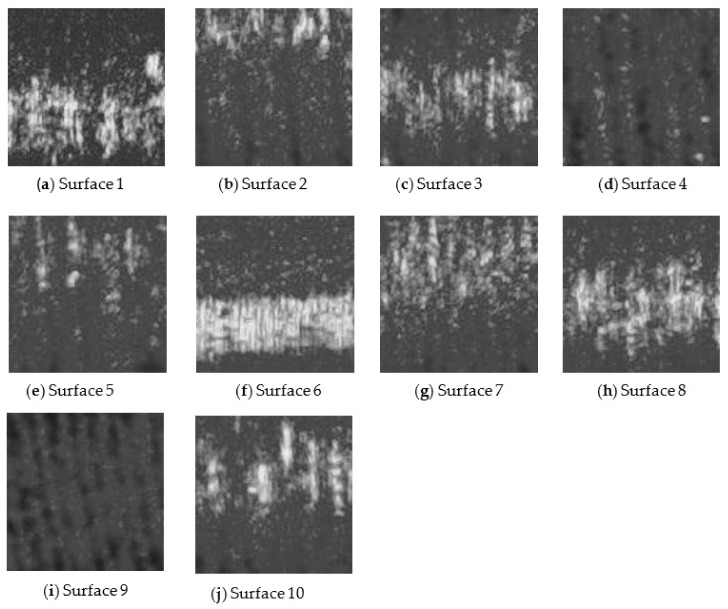
(**a**–**j**) Grayscale images of the laser speckle pattern image for each surface at an illumination angle of 45°, *f*-number of 16, and shutter speed of 1/100 s.

In the case of experimental setup 2, using MATLAB 2021a software, the speckle pattern image was cropped to a size of 101 × 101 pixels (maximum possible size) and converted from an RGB image to a grayscale image. Figure 7 shows the cropped grayscale image for the speckle pattern obtained for surfaces 1 to 10 at an illumination angle of 45°, *f*-number of 16, and shutter speed of 1/100 s for experimental setup 2. 

The grayscale image was not subjected to any filtering process to avoid the loss of data caused by filtering. Characteristic features based on the histogram, such as mean intensity, root-mean-square intensity, energy, entropy, and texture-based parameters, such as normalized roughness, and gray level co-occurrence matrix (GLCM)-based parameters, such as maximum probability, correlation, contrast, energy, homogeneity, and entropy, were extracted from the grayscale images. To differentiate the energy and entropy descriptors that are obtained from histogram-based and GLCM-based parameters, energy and entropy descriptors based on GLCM shall be addressed as energy (GLCM) and entropy (GLCM). From the binary image, the white-to-black pixels ratio was obtained as a characteristic feature. Coefficients of determination (R^2^) from the correlation study between the extracted characteristic features and 3D surface roughness were evaluated.

Equations (1)–(12) [10,27] that were used to extract the characteristic features from the image are as follows:

- Histogram-based (statistical) features
  ○ Mean

Mean of the gray value of the image *m* obtained from original image *f(x,y)* of size M × N, given by Equation (1).

(1) $$m=\frac{1}{MN}\overset{M-1}{\underset{x=0}{\sum }}\overset{N-1}{\underset{y=0}{\sum }}f\left(x,y\right)$$

where *f*(*x,y*) is the gray value of the pixel at coordinate (*x,y*).

○ Standard deviation

The standard deviation *σ* of an image is given by Equation (2).

(2) $$\sigma =\sqrt{\overset{L-1}{\underset{j=0}{\sum }}{\left(r_{j}-m\right)}^{2}p\left(r_{j}\right)}$$

where

*r_j_* is the *j*th gray level;
*L* is the total possible gray level value;
*p*(*r_j_*) is the probability of occurrences of *r_j_*;
*m* is the mean of gray values of the image.
  ○ Energy

The energy descriptor, which is also known as uniformity, measures how pixel values are distributed, along with the gray level range, and can be calculated for the grayscale image using Equation (3).

(3) $$energy=\overset{L-1}{\underset{j=0}{\sum }}{\left[p\left(r_{j}\right)\right]}^{2}\;$$

where

*r_j_* is the *j*th gray level;
*L* is the total possible gray level value;
*p*(*r_j_*) is the probability of occurrences of *r_j_.*
  ○ Entropy

The entropy descriptor provides information about the complexity of the image, as given by Equation (4).

(4) $$entropy=-\overset{L-1}{\underset{j=0}{\sum }}p\left(r_{j}\right)log_{2}\left[p\left(r_{j}\right)\right]\;$$

where

*r_j_* is the *j*th gray level;
*L* is the total possible gray level value;
*p*(*r_j_*) is the probability of occurrences of *r_j_*.

- Texture features
  ○ The normalised descriptor of roughness

The normalised descriptor of roughness *R* is as given in Equation (5).

(5) $$R=1-\frac{1}{1+\frac{\sigma^{2}}{{\left(L-1\right)}^{2}}}$$

where

*σ^2^* is variance;
*L* is the total possible gray level value.

- Gray level co-occurrence matrix (GLCM)

Histogram-based texture descriptors do not provide any information about the spatial relationship among pixels. This information can be obtained using the gray level co-occurrence matrix (GLCM). The matrix holds the information of the number of times pixels with intensities *r_i_* and *r_j_* occur in image *f*(*x,y*) in the position specified by the displacement vector (distance between two pixels *d,* and angle between the two pixels from horizontal, *θ*). In this work, as in the MATLAB software, default values of the displacement vector and orientation of *d* = 1 and *θ* = 0° were used. The matrix is normalized as given in Equation (6).

(6) $$\;N_{g}\;\left(i,j\right)=\frac{g\left(i,j\right)}{\sum_{i}\sum_{j}g\left(i,j\right)}$$

where

*N_g_*(*i,j*) is the normalized gray level co-occurrence matrix;
*g*(*i,j*) is the element of the gray level co-occurrence matrix.

The following texture-based features are computed using a normalized GLCM, *N_g_*(*i,j*).

○ Maximum probability (GLCM) is given by Equation (7).

(7) $$Maximum\;probability\;\left(GLCM\right)=max\;N_{g}\left(i,j\right)$$

○ Correlation (GLCM) is given by Equation (8).

(8) $$Correlation\;\left(GLCM\right)=\frac{\sum_{i}\sum_{j}\left(i-\mu_{i}\right)\left(j-\mu_{j}\right)N_{g}\left(i,j\right)}{\sigma_{i}\sigma_{j}}$$

where

*µ_i_* is the mean of the row sums of *N_g_*(*i,j*);
*µ_j_* is the mean of column sums of *N_g_*(*i,j*);
*σ_i_* is the standard deviation of row sums of *N_g_*(*i,j*);
*σ_j_* is the standard deviation of column sums of *N_g_*(*i,j*).

○ Contrast (GLCM) is given by Equation (9).
  (9) $$Contrast\;\left(GLCM\right)=\underset{i}{\sum }\underset{j}{\sum }{\left(i-j\right)}^{2}N_{g}\left(i,j\right)$$
○ Energy (GLCM) is given by Equation (10).
  (10) $$Energy\;\left(GLCM\right)=\underset{i}{\sum }\underset{j}{\sum }N_{g}^{2}\left(i,j\right)$$
○ Homogeneity (GLCM) is given by Equation (11).
  (11) $$Homogeneity\;\left(GLCM\right)=\underset{i}{\sum }\underset{j}{\sum }\frac{N_{g}\left(i,j\right)}{1+\left|i-j\right|}$$
○ Entropy (GLCM) is given by Equation (12).
  (12) $$Entropy\;\left(GLCM\right)=-\underset{i}{\sum }\underset{j}{\sum }N_{g}\left(i,j\right)log_{2}N_{g}\left(i,j\right)$$

- From the binary image, the following characteristic features were extracted:
  ○ Total white pixels to total black pixels ratio (W/B).

## 3. Results and Discussion

**Figure 8:**
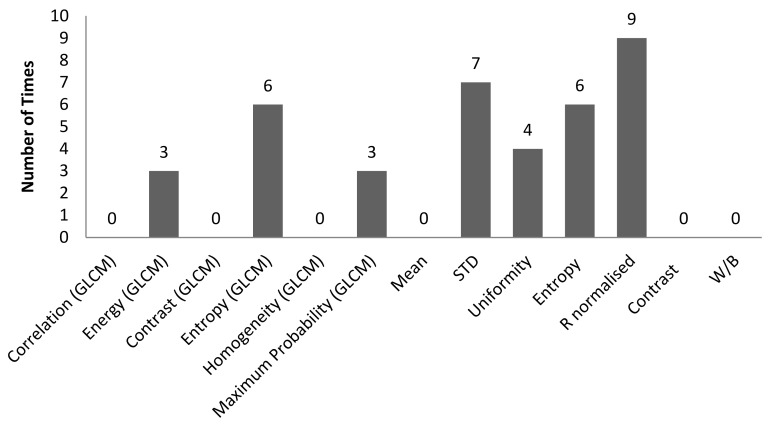
Bar chart showing the number of times a characteristic feature was involved in a correlation with R^2^ ≥ 0.7 for an illumination angle of 30° for experimental setup 1.

**Figure 9:**
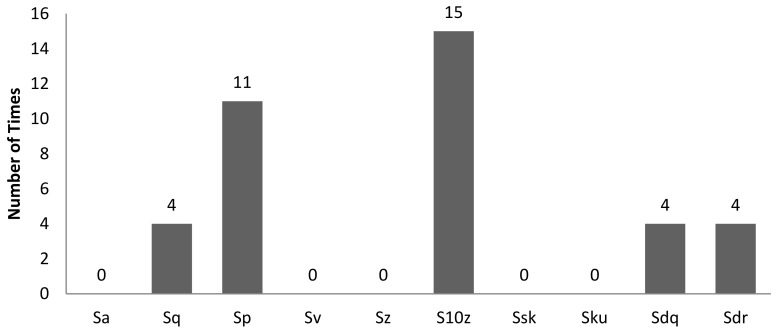
Bar chart showing the number of times a 3D surface roughness parameter was involved in correlation with R^2^ ≥ 0.7 for an illumination angle of 30° for experimental setup 1.

**Figure 10:**
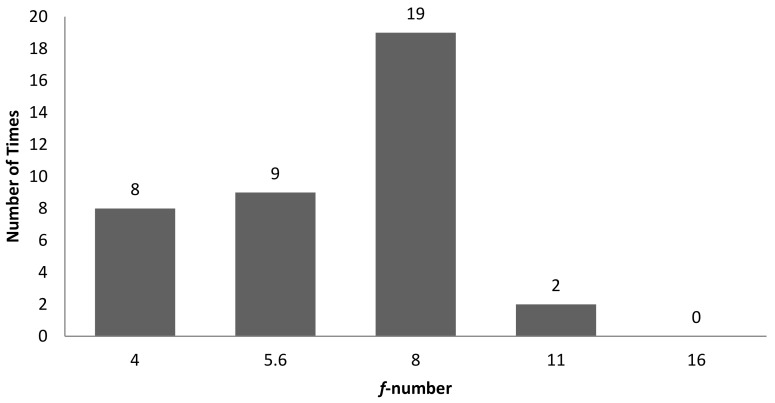
Bar chart showing the number of times there was correlation with R^2^ ≥ 0.7 for each *f*-number setting, for an illumination angle of 30° for experimental setup 1.

Table 2 is the tabulation of R^2^ ≥ 0.7 for an illumination angle of 30° for experiment setup 1. There are 38 correlations with an R^2^ ≥ 0.7. GLCM characteristic features account for 11 of the 38 correlations, while non-GLCM characteristic features account for 27 of the 38. Figure 8 shows the bar chart of the number of times a characteristic feature correlates with R^2^ ≥ 0.7. A characteristic feature, R normalised, had 9 out of 38 correlations with R^2^ ≥ 0.7. Figure 9 shows the bar chart of the number of times 3D surface roughness correlates with R^2^ ≥ 0.7. The 3D roughness parameter *S_10z_* had 15 out of 38 correlations. Figure 10 shows the bar chart of the number of times correlations with R^2^ ≥ 0.7 occurs for each *f*-number setting. The camera setting with *f*-number 8 had 19 correlations with R^2^ ≥ 0.7. Maximum probability (GLCM) vs. *S_dr_* had the highest R^2^ of 0.8742.

**Figure 11:**
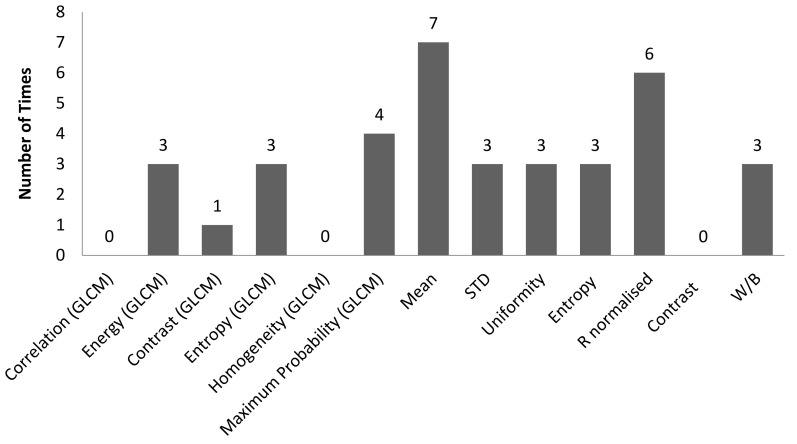
Bar chart showing the number of times a characteristic feature involved in correlation with R^2^ ≥ 0.7 for an illumination angle of 45° for experimental setup 1.

**Figure 12:**
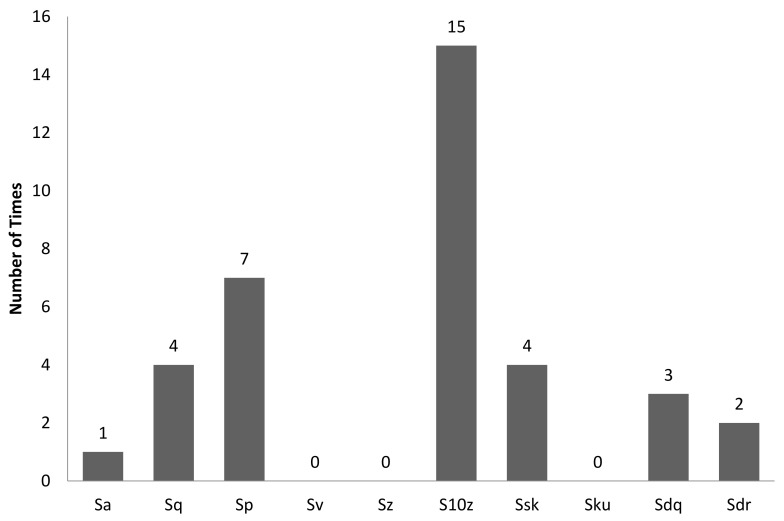
Bar chart showing the number of times a 3D surface roughness parameter was involved in correlation with R^2^ ≥ 0.7 for an illumination angle of 45° for experimental setup 1.

**Figure 13:**
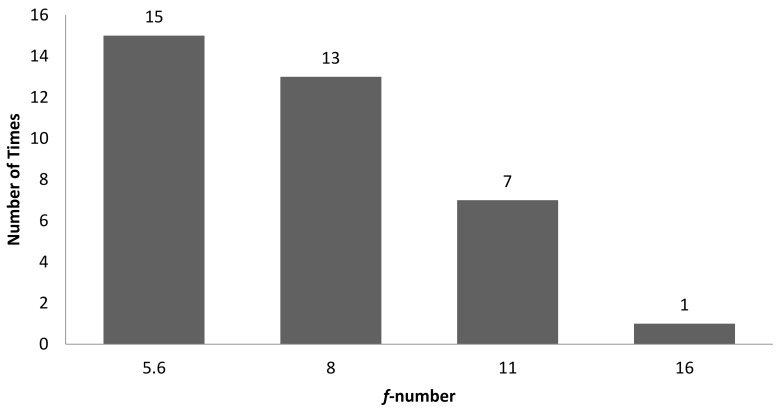
Bar chart showing the number of times correlation with R^2^ ≥ 0.7 occurred for each *f*-number setting for an illumination angle of 45° for experimental setup 1.

Table 3 is the tabulation of R^2^ ≥ 0.7 for an illumination angle of 45° for experiment setup 1. It was found that there were 36 correlations with R^2^ ≥ 0.7. Of these, 11 out of 36 correlations were GLCM characteristic features, and 25 out of 36 correlations were non-GLCM characteristic features. Figure 11 shows the bar chart of the number of times a characteristic feature correlates with R^2^ ≥ 0.7. The mean characteristic feature had 7 out of 36 correlations with R^2^ ≥ 0.7. Figure 12 shows a bar chart of the number of times 3D surface roughness correlates with R^2^ ≥ 0.7. The 3D roughness parameter S_10z_ had 15 out of 36 correlations. Figure 13 shows a bar chart of the number of times correlation with R^2^ ≥ 0.7 occurs for each *f*-number setting. The camera setting with *f*-number 5.6 had 15 correlations with R^2^ ≥ 0.7. The highest R^2^ was for maximum probability (GLCM) vs. S_dq_, with R^2^ = 0.9297.

**Figure 14:**
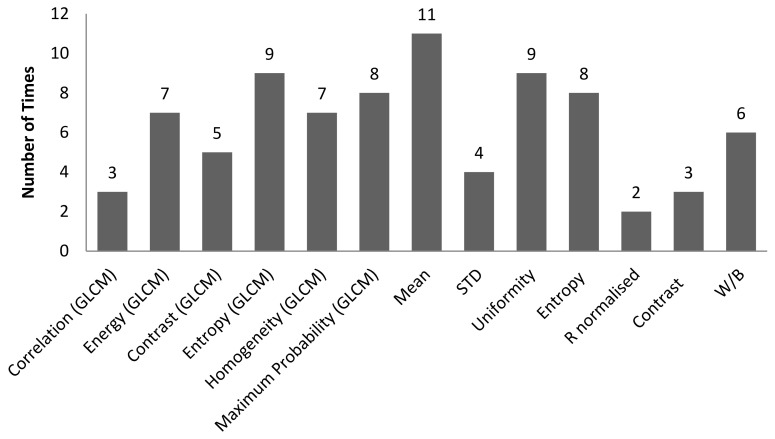
Bar chart showing the number of times a characteristic feature was involved in correlation with R^2^ ≥ 0.7 for an illumination angle of 60° for experimental setup 1.

**Figure 15:**
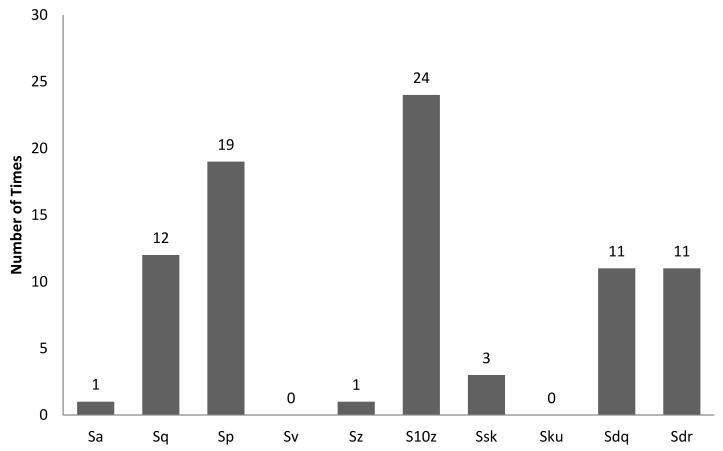
Bar chart showing the number of times a 3D surface roughness parameter was involved in correlation with R^2^ ≥ 0.7 for an illumination angle of 60° for experimental setup 1.

**Figure 16:**
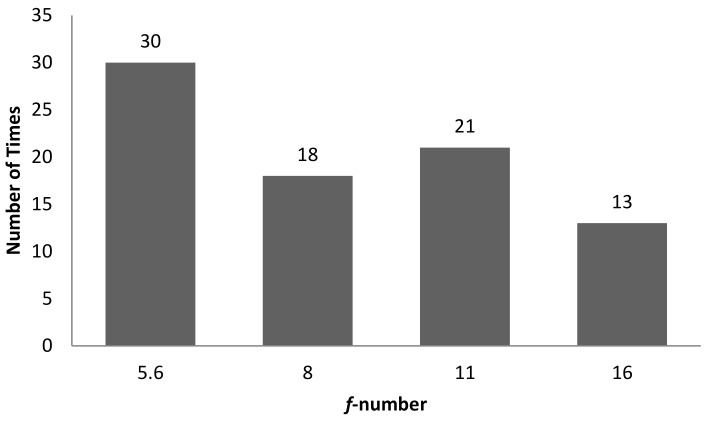
Bar chart showing the number of times correlation with R^2^ ≥ 0.7 occurred for each *f*-number setting for an illumination angle of 60° for experimental setup 1.

Table 4 is the tabulation of the number of times a combination of correlation between characteristic features and 3D surface roughness with R^2^ ≥ 0.7 occurred for an illumination angle of 60° for experiment setup 1. It was found that there were a total of 82 correlations with R^2^ ≥ 0.7. Of these, 39 out of 82 correlations were with GLCM characteristic features, while 43 out of 82 correlations were with non-GLCM characteristic features. Figure 14 shows the bar chart of the number of times a characteristic feature correlates with R^2^ ≥ 0.7. The mean characteristic feature had 11 out of 82 correlations with R^2^ ≥ 0.7. Figure 15 shows the bar chart of the number of times 3D surface roughness had a correlation with R^2^ ≥ 0.7. The 3D roughness parameter *S_10z_* had 24 out of 82 correlations with R^2^ ≥ 0.7. Figure 16 shows the bar chart of the number of times correlations with R^2^ ≥ 0.7 occurs for each *f*-number setting. The camera setting with *f*-number 5.6 had 30 correlations with R^2^ ≥ 0.7. The highest R^2^ value was for maximum probability (GLCM) vs. *S_dr_*, with R^2^ = 0.9384.

Table 5 is the tabulation of R^2^ ≥ 0.7 for an illumination angle of 30° for experiment setup 2. It was found that there were two correlations with R^2^ ≥ 0.7 at the camera setting with an *f*-number of 8 and shutter speed of 1/200 *s*. There was no correlation for non-GLCM characteristic features. The only characteristic features with correlation were correlation (GLCM) and roughness parameters *S_a_* and *S_q_*, each having a correlation of 1 out of 2. The highest R^2^ was for correlation (GLCM) vs. *S_q_*, with R^2^ = 0.7438.

Table 6 is the tabulation of R^2^ ≥ 0.7 for an illumination angle of 45° for experimental setup 2. It was found that there were 19 correlations with R^2^ ≥ 0.7. An *f*-number of 8 with a shutter speed of 1/50 s had two correlations with R^2^ ≥ 0.7. An *f*-number of 16 with a shutter speed 1/100 s had eight correlations with R^2^ ≥ 0.7. An *f*-number of 22 with a shutter speed of 1/100 s had two correlations with R^2^ ≥ 0.7. An *f*-number of 22 with a shutter speed 1/200 s had seven correlations with R^2^ ≥ 0.7. In total, 13 out of 19 correlations were GLCM characteristic features, and 6 out of 19 correlations were non-GLCM characteristic features. The correlation (GLCM) characteristic feature had 4 out 19 correlations, and roughness parameters *S_p_* and *S_z_* each had 4 out of 19 correlations. The highest R^2^ is for energy (GLCM) vs. *S_10z_*, with R^2^ = 0.8955.

Experimental setup 1 had correlations with R^2^ ≥ 0.7 at illumination angles of 30°, 45°and 60°, whereas experimental setup 2 had correlations with R^2^ ≥ 0.7 only at illumination angles of 30° and 45°. In the case of experimental setup 1, there were more non-GLCM characteristic features compared with GLCM characteristic features. The mean characteristic feature had more correlations with R^2^ ≥ 0.7 for illumination angles of 45° (7 out of 36 correlations) and 60° (11 out of 82 correlations), and R-normalized had more correlations with R^2^ ≥ 0.7 in the case of illumination angles of 30° (9 out of 38 correlations). Roughness parameter *S_10z_* had more correlation with R^2^ ≥ 0.7 for all of the illumination angles (15 out of 38 correlations for angle 30°, 15 out of 36 correlations for angle 45°, and 24 out of 82 correlations for angle 60°) in the experimental setup 1. For experimental setup 2, correlation (GLCM) characteristic features had more correlations with R^2^ ≥ 0.7 (2 out of 2 correlations for an illumination angle of 30°, and 4 out of 19 correlations for an illumination angle of 45°). Roughness parameters *S_a_* and *S_q_* had more correlations with R^2^ ≥ 0.7 for an illumination angle of 30 (1 out of 2 correlations each), and *S_p_* and *S_z_* had more correlations with R^2^ ≥ 0.7 for an illumination angle of 45 (4 out of 19 correlations each) for experimental setup 2. The reason for better correlation using experimental setup 1 compared to experimental setup 2 could be due to the expansion of the laser beam, which reduced the intensity of the laser beam that fell onto the surface. In this way, pixel saturation can be avoided. Another reason could be due to cleaning the laser beam of noise using a spatial filter. 

## 4. Conclusions

From the results, it can be seen that there are good correlations between characteristic features and 3D surface roughness in both the experimental setups. Experimental setup 1 gives a better correlation compared to experimental setup 2. In the case of experimental setup 1, all the illumination angles had correlations with R^2^ ≥ 0.7, and in the case of experimental setup 2, there were no correlations in the case of an illumination angle of 60°. The illumination angle of 60° gave the highest number of correlations with R^2^ ≥ 0.7 in the case of experimental setup 1 (82 correlations), and the illumination angle of 45° gave the highest number of correlations with R^2^ ≥ 0.7 in the case of experimental setup 2 (19 correlations). Mean characteristic features had more correlation with R^2^ ≥ 0.7 in the case of an angle of illumination of 45° and 60°, and R normalized characteristic features had more correlation with R^2^ ≥ 0.7 in the case of an angle of illumination of 30° for experimental setup 1. Correlation (GLCM) had more correlation with R^2^ ≥ 0.7 in the case of experimental setup 2. Roughness parameters *S_a_* and *S_q_* had more correlation with R^2^ ≥ 0.7 for an illumination angle of 30°, and *S_p_* and *S_z_* had more correlation with R^2^ ≥ 0.7 for an illumination angle of 45° in the case of experimental setup 2. A spatial filter that cleans the laser beam and then expands the cleaned beam could be the reason for the better result in the case of experimental setup 1. Previous works focus more on correlation studies involving 2D roughness parameters, while current work is focused on the correlation involving 3D surface roughness and characteristic features extracted from the laser speckle pattern. The current work shows that although experimental setup 1 gives better results compared to experimental setup 2, experimental setup 2 uses inexpensive components and is simple compared to experimental setup 1, and there is room for improvement in future work whereby the experimental setup 2 should be carried out by replacing the DSLR camera with a webcam. The novelty of this work is that it is (1) a correlation study between 3D surface roughness and speckle pattern using a He-Ne laser and spatial filter; and (2) a comparison study between two experimental setups on the correlation between 3D surface roughness and speckle pattern.

## Figures and Tables

**Table 1:** Tabulation of machining parameters and 3D roughness parameters of each surface [10].

Surface No.	Spindle Speed (rpm)	Feed Rate (mm/min)	Depth of Cut (mm)	*S_a_* (µm)	*S_q_* (µm)	*S_p_* (µm)	*S_v_* (µm)	*S_z_* (µm)	*S_10z_* (µm)	*S_sk_*	*S_ku_*	*S_dq_*	*S_dr_* (%)
1	1000	120	1	0.931	1.117	4.070	3.889	7.959	6.187	−0.044	2.415	0.161	1.305
2	1000	280	1	1.046	1.322	8.081	7.774	15.855	9.217	0.281	3.398	0.177	1.553
3	1000	440	1	1.325	1.675	9.809	6.795	16.605	10.826	0.146	3.442	0.191	1.790
4	1000	600	1	1.162	1.567	11.639	13.136	24.775	14.423	0.275	6.054	0.242	2.636
5	1000	760	1	1.254	1.816	10.948	8.678	19.626	16.608	0.360	6.142	0.318	4.565
6	2500	120	1	0.748	0.902	5.681	6.960	12.641	5.122	0.335	2.473	0.185	1.722
7	2500	280	1	0.968	1.156	5.437	8.607	14.045	6.387	−0.177	6.528	0.185	1.749
8	2500	440	1	0.974	1.225	7.169	6.327	13.496	7.440	−0.124	4.374	0.196	1.830
9	2500	600	1	1.058	1.326	8.617	9.490	18.106	8.325	0.000	3.152	0.192	1.888
10	2500	760	1	1.113	1.417	9.640	6.377	16.016	10.246	0.257	3.715	0.193	1.811

**Table 2:** Tabulation of the number of times a correlation between characteristic features and 3D surface roughness occurs with R^2^ ≥ 0.7 for an illumination angle of 30° for experimental setup 1.

Characteristic Features	3D Surface Roughness Parameters									
	*S_a_*	*S_q_*	*S_p_*	*S_v_*	*S_z_*	*S_10z_*	*S_sk_*	*S_ku_*	*S_dq_*	*S_dr_*
Correlation (GLCM).										
Energy (GLCM)		1	1			1				
Contrast (GLCM)										
Entropy (GLCM)		1	3			2				
Homogeneity (GLCM)										
Maximum probability (GLCM)			1						1	1
Mean										
STD			1			4			1	1
Uniformity		1	1			2				
Entropy		1	3			2				
R normalised			1			4			2	2
Contrast										
W/B										

**Table 3:** Tabulation of the number of times a correlation between characteristic features and 3D surface roughness occurs with R^2^ ≥ 0.7 for an illumination angle of 45° for experimental setup 1.

Characteristic Features	3D Surface Roughness Parameters									
	*S_a_*	*S_q_*	*S_p_*	*S_v_*	*S_z_*	*S_10z_*	*S_sk_*	*S_ku_*	*S_dq_*	*S_dr_*
Correlation (GLCM)										
Energy (GLCM)			1						1	1
Contrast (GLCM)						1				
Entropy (GLCM)			1			2				
Homogeneity (GLCM)										
Maximum probability (GLCM)		1				1			1	1
Mean		2	1			4				
STD			1				2			
Uniformity			1			2				
Entropy			1			2				
R normalised			1			2	2		1	
Contrast										
W/B	1	1				1				

**Table 4:** Tabulation of number of times a correlation between characteristic features and 3D surface roughness occurs with R^2^ ≥ 0.7 for an illumination angle of 60° for experimental setup 1.

Characteristic Features	3D Surface Roughness Parameters									
	*S_a_*	*S_q_*	*S_p_*	*S_v_*	*S_z_*	*S_10z_*	*S_sk_*	*S_ku_*	*S_dq_*	*S_dr_*
Correlation (GLCM)						1			1	1
Energy (GLCM)		1	2			2			1	1
Contrast (GLCM)		1	1			1			1	1
Entropy (GLCM)		1	3			3			1	1
Homogeneity (GLCM)		2	1			2			1	1
Maximum probability (GLCM)		1	3		1	1			1	1
Mean		2	4			3			1	1
STD		1				1	2			
Uniformity		1	2			3	1		1	1
Entropy		1	3			3				1
R normalised						1			1	
Contrast	1	1				1				
W/B						2			2	2

**Table 5:** Tabulation of R^2^ ≥ 0.7 for an illumination angle of 30° for experimental setup 2.

S. No.	Correlation	R^2^	Camera Setting
1	Correlation (GLCM) vs. *S_a_*	0.7354	*f*-number 8, shutter speed 1/200 s
2	Correlation (GLCM) vs. *S_q_*	0.7438	

**Table 6:** Tabulation of R^2^ ≥ 0.7 for an illumination angle of 45° for experimental setup 2.

S. No.	Correlation	*R* ^2^	Camera Setting
1	Entropy (GLCM) vs. *S_a_*	0.8208	*f*-number 8, shutter speed 1/50 s
2	Entropy (GLCM) vs. *S_q_*	0.7352	
3	Energy vs. *S_p_*	0.7347	*f*-number 16, shutter speed 1/100 s
4	Energy (GLCM) vs. *S_p_*	0.7202	
5	Energy vs. *S_z_*	0.7015	
6	Entropy vs. *S_z_*	0.7354	
7	Entropy (GLCM) vs. *S_z_*	0.7565	
8	Homogeneity (GLCM) vs. *S_z_*	0.7704	
9	Energy vs. *S*_10z_	0.8916	
10	Energy (GLCM) vs. *S*_10z_	0.8955	
11	W/B vs. *S_dq_*	0.8151	*f*-number 22, shutter speed 1/100 s
12	W/B vs. *S_dr_*	0.8294	
13	Contrast (GLCM) vs. *S_a_*	0.749	*f*-number 22, shutter speed 1/200 s
14	Correlation (GLCM) vs. *S_a_*	0.806	
15	Contrast (GLCM) vs. *S_q_*	0.7358	
16	Correlation (GLCM) vs. *S_q_*	0.8148	
17	Contrast (GLCM) vs. *S_p_*	0.7368	
18	Correlation (GLCM) vs. *S_p_*	0.8403	
19	Correlation (GLCM) vs. *S*_10z_	0.7316	

## Data Availability

Not applicable.
